# Development and validation of a skin health knowledge, attitudes, and behaviors questionnaire for the general population: a cross-sectional study

**DOI:** 10.3389/fpubh.2026.1856843

**Published:** 2026-06-08

**Authors:** Xinquan Wang, Liping Jin, Yuanzhi Wang, Wenhao Fu, Minxue Shen, Wu Zhu

**Affiliations:** 1 Department of Dermatology, Hainan General Hospital (Hainan Affiliated Hospital of Hainan Medical University), Haikou, Hainan, China; 2 Department of Dermatology, Hunan Engineering Research Center of Skin Health and Disease, Hunan Key Laboratory of Skin Cancer and Psoriasis, Xiangya Hospital, Central South University, Changsha, China; 3 Department of Social Medicine and Health Management, Xiangya School of Public Health, Central South University, Changsha, China

**Keywords:** confirmatory factor analysis, content validity index, cross-sectional survey, item response theory, Knowledge–Self-Efficacy–Attitudes–Behaviors, skin health questionnaire

## Abstract

**Background:**

Existing skin health assessments predominantly focus on single topics or diseases, making it difficult to cover the complete “Knowledge–Self-efficacy–Attitudes–Behaviors (KSAB)” continuum.

**Objective:**

To develop and validate a skin health knowledge, attitudes, and behaviors questionnaire for the general population.

**Methods:**

A cross-sectional online survey was conducted among the general population between October and November 2025. The questionnaire was developed based on a conceptual structure incorporating four dimensions: knowledge, self-efficacy, attitudes, and behaviors. Content validity was assessed by 13 experts, and the Item-level Content Validity Index (I_CVI_) and modified Kappa (K^*^) were calculated. Construct validity was examined using confirmatory factor analysis (CFA). Item-level evaluation was performed using item response theory (IRT), including the two-parameter logistic (2PL) model for dichotomous items and the graded response model (GRM) for ordered items.

**Results:**

A total of 865 individuals were enrolled (men: women = 1.24:1). Content validity was good (I_CVI_ = 0.846–1.000, K* = 0.845–1.000). The five-factor model from CFA showed a good fit (CFI = 0.969, TLI = 0.960, RMSEA = 0.070, SRMR = 0.095). IRT analysis indicated that K1–K5 had acceptable discrimination (*a* = 0.59–2.40) and difficulty parameters ranging from −3.63 to −1.33. However, items K6, K7 and S1 were excluded due to insufficient threshold coverage. The proportion of women reporting frequent sunscreen use was higher than that of men. Self-efficacy levels showed a decreasing trend with age. Furthermore, individuals in service/manual labor occupations exhibited lower proportions on certain items.

**Conclusion:**

This study developed and validated a KSAB questionnaire on skin health knowledge, attitudes, and behaviors, showing good validity and item-level performance. The findings support targeted health promotion for men, older adults, and service/manual workers.

## Background

Skin health is closely associated with an individual’s quality of life. Common skin issues not only affect appearance and psychosocial function but may also impose long-term health and economic burdens (1, 2). In addition to genetic and physiological factors, lifestyle and skincare behaviors play a crucial role in maintaining skin health and preventing risks (3, 4). Notably, factors such as sun exposure are strongly linked to various adverse skin-related outcomes, underscoring the profound public health significance of enhancing public sun protection behaviors and related knowledge (5). Previous studies from Western populations have reported inadequate skin care habits, chronic sun exposure, and insufficient photo-protection awareness, highlighting the need for educational interventions and skin health related assessment across different populations (6, 7). The promotion of skin health depends not only on the level of knowledge but also on an individual’s ability to access and filter reliable information, understand and discern media/advertising claims, develop scientific attitudes, and translate these into consistent behaviors (8). Therefore, assessing skin health knowledge, self-efficacy, attitudes, and behaviors using a multidimensional conceptual structure incorporating these domains (KSAB) may help systematically identify areas of weakness, thereby providing a basis for health education, risk communication, and the development of intervention strategies (9, 10).

Currently, measurement tools related to skin health predominantly focus on single topics or specific contexts, such as assessing only sun protection behaviors or specific conditions like atopic dermatitis and skin cancer (9, 11–13). These tools exhibit certain limitations in content coverage, structural consistency, and applicability across diverse populations. To develop a comprehensive assessment instrument suitable for the Chinese population, this study developed and validated the Skin Health Knowledge, Attitudes, and Behaviors Questionnaire. We evaluated the questionnaire’s content relevance, underlying construct structure, and item-level measurement performance, and further explored variations in responses across key demographic subgroups. This process provides psychometric evidence and informs potential directions for public health interventions using this questionnaire.

## Method

### Study design and participants

This study was a cross-sectional survey designed to develop and validate the Skin Health Knowledge, Attitudes, and Behaviors Questionnaire. The questionnaire was developed based on a conceptual structure incorporating knowledge, self-efficacy, attitudes, and behaviors. The survey was conducted between October 2025 and November 2025, with participants recruited via an online platform. All participants were eligible for inclusion except those who submitted invalid questionnaires. This study involved an anonymous online survey, and no directly identifiable personal information was collected. All participants read the online informed consent statement and voluntarily agreed to participate before completing the questionnaire, in accordance with the Declaration of Helsinki (Supplementary Appendix S1).

### Questionnaire design

The questionnaire was developed based on a conceptual structure incorporating knowledge, self-efficacy, attitudes, and behaviors. Relevant literature on KAP-related questionnaires, self-efficacy theory, and health literacy was reviewed to inform domain identification and item development (8, 10, 13). An initial item pool was generated through discussions within the research team. Items were designed to cover the predefined domains and were subsequently refined to improve clarity, consistency, and relevance while reducing ambiguity and redundancy. The revised questionnaire was then evaluated through expert consultation and content validity assessment before finalization. No formal cognitive interviews or pilot testing procedures were conducted prior to the survey. An initial item pool was developed through discussions within the research team. The final questionnaire comprised 26 items across 5 sections and 4 dimensions, covering: (1) Baseline data collection (gender, age, occupation, annual household income, and history of diagnosed skin diseases). Household annual income categories were defined by the research team based on household income levels in China at the time of survey administration (2025); (2) Knowledge dimension (true/false items and Likert-scale items on misconceptions related to skin care); (3) Self-efficacy dimension (perceived ability to access, understand, evaluate, and apply skin health information); (4) Attitude dimension (perception of the importance of skin care and proactive engagement); and (5) Behavior (skin care practices and information-seeking behaviors). The initial items underwent wording revision and consistency checks by the research team to ensure clarity, avoid ambiguity, and reduce redundancy, resulting in the versions used for expert consultation and the formal survey (Supplementary Table S1).

### Content validity assessment

Content validity was evaluated through expert consultation involving 13 experts, including dermatologists and experts in biostatistics and questionnaire methodology. Experts were invited to rate the relevance of each item using a 4-point Likert scale: 1 = not relevant, 2 = somewhat not relevant, 3 = somewhat relevant, 4 = highly relevant. Experts evaluated item relevance, clarity, comprehensiveness, and representativeness. Following common methodology for calculating the content validity index (CVI), ratings were dichotomized: scores of 3–4 were classified as “relevant” and scores of 1–2 as “not relevant” (14). The item-level content validity index (I_CVI_) was defined as the proportion of experts rating an item as “relevant” among the total number of experts. Items with an I_CVI_ ≥ 0.78 were considered acceptable (14, 15). To correct for chance agreement, the probability of chance agreement (P_c_) and the modified Kappa statistic (K^*^) were further calculated using the following formulas:


$$P_{c}=\frac{N!}{A!(N-A)!}\times 0.5^{N};K^{*}=(I_{\mathrm{CVI}}-P_{c})/(1-P_{c})$$

Where *N* is the total number of experts and *A* is the number of experts rating the item as “relevant.” Items with relatively low I_CVI_ values were revised based on expert feedback before finalization of the questionnaire.

### Data entry

Data were collected online and subsequently exported. Responses were coded (X.W), and open-ended text items were entered verbatim. A second statistician (M.S) verified the entries. A quality control item (QC-K) was included in the questionnaire to assess participant attentiveness. In the dataset for this study, this item was consistently answered as “No” in accordance with the instruction, indicating acceptable overall data quality.

### Scoring and data processing

Scale items were scored according to predefined scoring rules. Specifically: (1) For dichotomous knowledge judgment items: correct = 1 point, incorrect = 0 points; (2) For misconception-type Likert items: a 5-point agreement scale was used and scored in the direction of “higher disagreement with the erroneous belief yielding a higher score” (reverse scoring was applied when necessary), so that a higher score represented more accurate knowledge/more reasonable cognition; (3) For self-efficacy and attitude items: a 5-point agreement scale was used with positive scoring, so that a higher score represented greater self-efficacy/more positive attitude.

For IRT analysis, dichotomous items were coded as 0/1, and Likert-scale and other ordinal-scored items were treated as ordered polytomous variables (16).

### Item response theory (IRT) analysis

To evaluate the psychometric performance at the item level and to select acceptable items, this study employed IRT to estimate item parameters: (1) For dichotomous knowledge items, the two-parameter logistic (2PL) model was used to estimate the discrimination parameter *a* and the difficulty parameter *b*. The discrimination parameter *a* reflects an item’s ability to differentiate the latent trait (*θ*), while the difficulty parameter *b* represents the *θ* level at which the probability of a correct response is 0.50. (2) For polytomous ordered items, the graded response model (GRM) was applied to estimate the discrimination parameter *a* and the threshold parameters *b*_1_ to *b*_k_. Items with parameters *a* ≥ 0.5 and mean |*b*_k_| < 3 were acceptable. All analyses were conducted in R (version 4.3.2).

### Structural validity assessment

CFA was employed to assess the structural validity of the scale (17). Given the mixed item formats (dichotomous and ordinal responses), all items were treated as ordered categorical indicators and the model was estimated using the weighted least squares mean and variance adjusted (WLSMV) estimator. A five-factor model was specified, with the Knowledge domain modeled as two correlated subdomains: one for the factual true/false items (K1–K5) and another for the misconception-related Likert items (K6–K7), together with the Self-efficacy (S1–S4), Attitudes (A1–A2), and Behaviors (B1–B3) domains. We report the standardized factor loadings (*λ*) and their statistical significance. Model fit was assessed using the CFI, TLI, RMSEA, and SRMR indices.

### Data analysis

All statistical analyses were performed using R version 4.3.2. Descriptive analyses were employed to statistically analyze the questionnaire items. For true/false questions, the proportion of incorrect responses was calculated to reflect misconceptions, defined as the ratio of the number of incorrect responses to the number of valid respondents. For Likert-scale items, the frequency and percentage (%) of each response category were calculated. For multiple-choice questions, the selection proportion for each option (number of respondents selecting the option divided by the number of valid respondents) was calculated among valid respondents. All results were presented visually.

To compare the performance of populations with different baseline characteristics across items, item responses were dichotomized into “positive/correct” and “non-positive/incorrect” according to predefined rules. For true/false items, a “correct answer” was considered a correct response. For Likert-type items, responses were scored from 0 to 4, corresponding to “strongly disagree,” “disagree,” “not sure,” “agree,” and “strongly agree,” respectively. Accordingly, scores ≥3 were considered positive responses. Subsequently, for each subgroup defined by baseline variables (gender, age group, occupation, annual household income, and history of dermatological diagnosis), the proportion of positive/correct responses for each item (number of positive or correct respondents/number of valid respondents in that subgroup) was calculated. A heatmap was used to visualize the differences in proportions across subgroups for different items.

## Results

A total of 865 respondents were included in this study, comprising 478 men (55.3%) and 387 women (44.7%). Approximately half of the respondents reported a previous diagnosis of a dermatological condition (441 individuals, 51.0%), with acne (19.2%) and eczema (20.9%) being the most common. The distribution of annual household income was relatively dispersed (Table 1).

**Table 1:** Baseline characteristics of participants (*N* = 865).

Characteristics	*n* (%)
Gender	
Men	478 (55.3)
Women	387 (44.7)
Age group (years)	
<18	24 (2.8)
18–25	365 (42.2)
26–30	94 (10.9)
31–40	212 (24.5)
41–50	115 (13.3)
51–60	42 (4.9)
>60	13 (1.5)
Employment status	
Student	348 (40.2)
Company employee	226 (26.1)
Professional	102 (11.8)
Self-employed	51 (5.9)
Government/public sector employee	46 (5.3)
Technical worker	41 (4.7)
Retired	28 (3.2)
Service worker	14 (1.6)
Production worker	9 (1.0)
Annual household income (CNY)	
<20,000	185 (21.4)
20,000–49,000	134 (15.5)
50,000–79,000	120 (13.9)
80,000–119,000	125 (14.5)
120,000–230,000	138 (16.0)
>230,000	163 (18.8)
History of skin disease^*^	
No	424 (49.0)
Yes	441 (51.0)
Types of skin disease^**^	
Eczema	181 (20.9)
Acne	166 (19.2)
Urticaria	129 (14.9)
Cutaneous fungal infection	86 (9.9)
Hair loss (overall)	68 (7.9)
Alopecia areata	11 (1.3)
Unspecified	37 (4.3)
Androgenetic	18 (2.1)
Other Alopecia	2 (0.2)
Herpes zoster	31 (3.6)
Contact dermatitis	29 (3.4)
Viral warts	22 (2.5)
Scabies	20 (2.3)
Rosacea	17 (2.0)
Vitiligo	9 (1.0)
Psoriasis	9 (1.0)
Other skin conditions	14 (1.6)

* History of skin disease was self-reported (Yes/No).

** Types of skin disease: multiple responses allowed; percentages are calculated based on the total sample (*N* = 865). Bold values indicate items recommended for priority revision or removal in subsequent scale optimization.

Thirteen experts rated the relevance of the items. Overall, the content validity of the questionnaire items was satisfactory: the I-CVI ranged from 0.846 to 1.000, with most items achieving an I-CVI of 1.000; the modified Kappa values ranged from 0.845 to 1.000, indicating high reliability in the assessment of item relevance (Supplementary Table S2).

This study employed confirmatory factor analysis (CFA) to examine the structural validity of the scale and a five-factor model was fitted. The overall model fit was good (CFI = 0.969, TLI = 0.960, RMSEA = 0.070, SRMR = 0.095; *χ*^2^(94) = 494.34, *p* < 0.001). Standardized factor loadings were generally high for the self-efficacy, behavior, attitude, and misconception factors (ranging from 0.60 to 0.82, 0.72–0.96, 0.75–0.89, and 0.79–0.85, respectively). Loadings for the factual knowledge factor ranged from 0.16 to 0.73 (Supplementary Table S3).

In this study, IRT was employed to evaluate the items at the project level. For the dichotomous true/false knowledge items (K1–K5), parameter estimation was performed using the 2PL model (Table 2). The results indicated that the discrimination parameter *a* ranged from 0.59 to 2.40, suggesting variability in the items’ ability to differentiate latent knowledge levels (*θ*). Among them, K2 exhibited the highest discrimination (*a* = 2.40), while K3 (*a* = 0.59) and K4 (*a* = 0.77) were relatively lower. Overall, this set of true/false items demonstrated low difficulty (*b* = −3.63 to −1.33), indicating that the items were generally easy for the present sample. For the polytomous Likert-scale items, the GRM was used to estimate the discrimination parameter *a* and the threshold parameters *b*_1_*–b*_4_ (Table 2). The discrimination of the items varied considerably (*a* = 0.34–7.14). Items in the self-efficacy dimension generally exhibited moderate to high discrimination (*a* = 0.93–3.07), with S2 (*a* = 2.55) and S3 (*a* = 3.07) being higher. Within the attitude dimension, A1 showed the highest discrimination (*a* = 6.51). Consequently, the knowledge misconception items K6 (*a* = 0.46) and K7 (*a* = 0.34) demonstrated insufficient discrimination, and their threshold distributions were skewed toward extremes. Additionally, the self-efficacy item S1 had a relatively large average absolute value for its thresholds, suggesting suboptimal coverage of the latent trait. The aforementioned items are recommended for priority revision or removal in subsequent scale optimization.

**Table 2:** Parameters estimated with item response models.

Model	Domain	Item	*a*	*b_1_*	*b_2_*	*b_3_*	*b_4_*
2PL	Knowledge	K1	1.27	−1.33			
	Knowledge	K2	2.40	−1.41			
	Knowledge	K3	0.59	−3.31			
	Knowledge	K4	0.77	−3.63			
	Knowledge	K5	1.00	−2.50			
GRM	**Knowledge**	**K6**	**0.46**	**−5.46**	**−2.63**	**1.61**	**5.89**
	**Knowledge**	**K7**	**0.34**	**−6.54**	**−1.12**	**3.48**	**8.74**
	Knowledge	K8	7.14	−1.80	−1.53		
	Knowledge	K9	1.77	−1.58	−0.05		
	Knowledge	K10	2.56	−1.76	−1.06		
	**Self-efficacy**	**S1**	**0.93**	**−6.51**	**−5.18**	**−3.59**	**−1.01**
	Self-efficacy	S2	2.55	−2.77	−1.86	−0.51	0.68
	Self-efficacy	S3	3.07	−2.47	−1.73	−0.16	0.83
	Self-efficacy	S4	1.27	−3.25	−1.95	−0.29	1.45
	Attitude	A1	6.51	−2.79	−2.33	−1.37	0.07
	Attitude	A2	1.74	−3.54	−2.27	−0.80	0.54

Item parameters were estimated using 2PL item and GRM. Discrimination (a) reflects how strongly an item differentiates respondents along the latent trait (*θ*). Thresholds (b_1_–b_4_) represent the trait levels at which the probability of responding in category k or higher equals 0.50.

**Figure 1:**
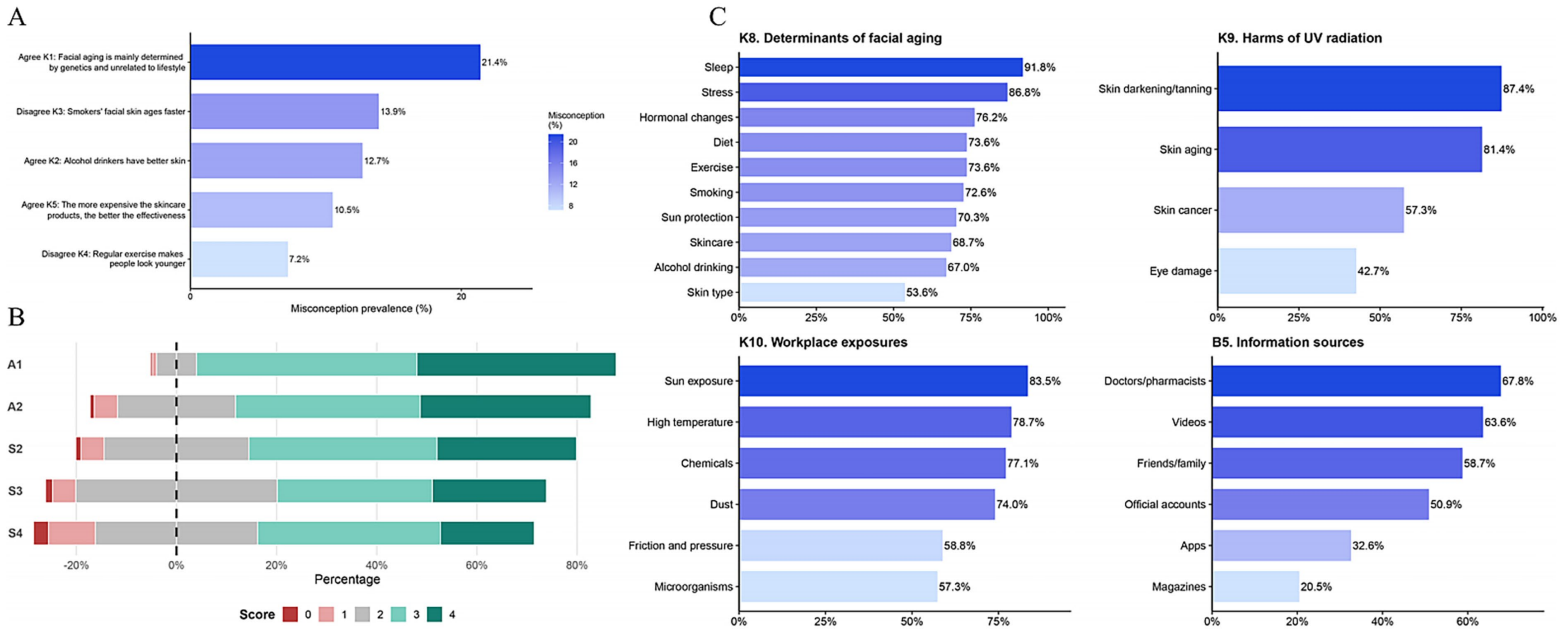
Item-level response distributions across KSAB domains in the Skin Health Behavior and Literacy Questionnaire (*N* = 865). **(A)** Prevalence of misconceptions for dichotomous knowledge items (K1–K5), calculated as the proportion of incorrect responses. **(B)** Response distributions for Likert-type items (0–4), where 0 = strongly disagree, 1 = disagree, 2 = not sure, 3 = agree, and 4 = strongly agree. Bars represent the percentage of participants selecting each response category. **(C)** Selection proportions for multiple-response items on skin health perceptions and information sources, including knowledge/perception items (K8–K10) and information channels (B5). Values represent the percentage of participants endorsing each option.

In the binary knowledge assessment items, the overall prevalence of misconceptions ranged from 7.2 to 21.4% (Figure 1A and Supplementary Table S4). Among these, statement K1 exhibited the highest prevalence of misconceptions (21.4%), while K4 had a relatively lower misconception rate (7.2%). Statistical analysis was performed on the proportion of responses at different levels for each Likert item (Figure 1B). The results indicated that the overall response distribution for all Likert items was predominantly skewed toward higher scores. Specifically, the response distributions for attitude items A1 and A2, as well as self-efficacy item S2, were biased toward more positive directions. In the multiple-choice questions (Figure 1C), respondents identified sleep quality (91.9%) and stress (86.7%) as the primary influencing factors for facial aging. Regarding awareness of ultraviolet radiation hazards, a higher proportion of respondents selected “skin tanning/darkening” (87.4%) and “skin aging” (81.4%), while the proportions selecting “skin cancer” (57.3%) and “eye damage” (42.7%) were relatively lower. Concerning occupational environmental exposures potentially affecting skin health, sun exposure (83.5%) and high temperatures (78.7%) were most frequently mentioned. Regarding information acquisition channels, doctors/pharmacists (67.8%) and video (63.6%) were identified as the primary sources.

**Figure 2:**
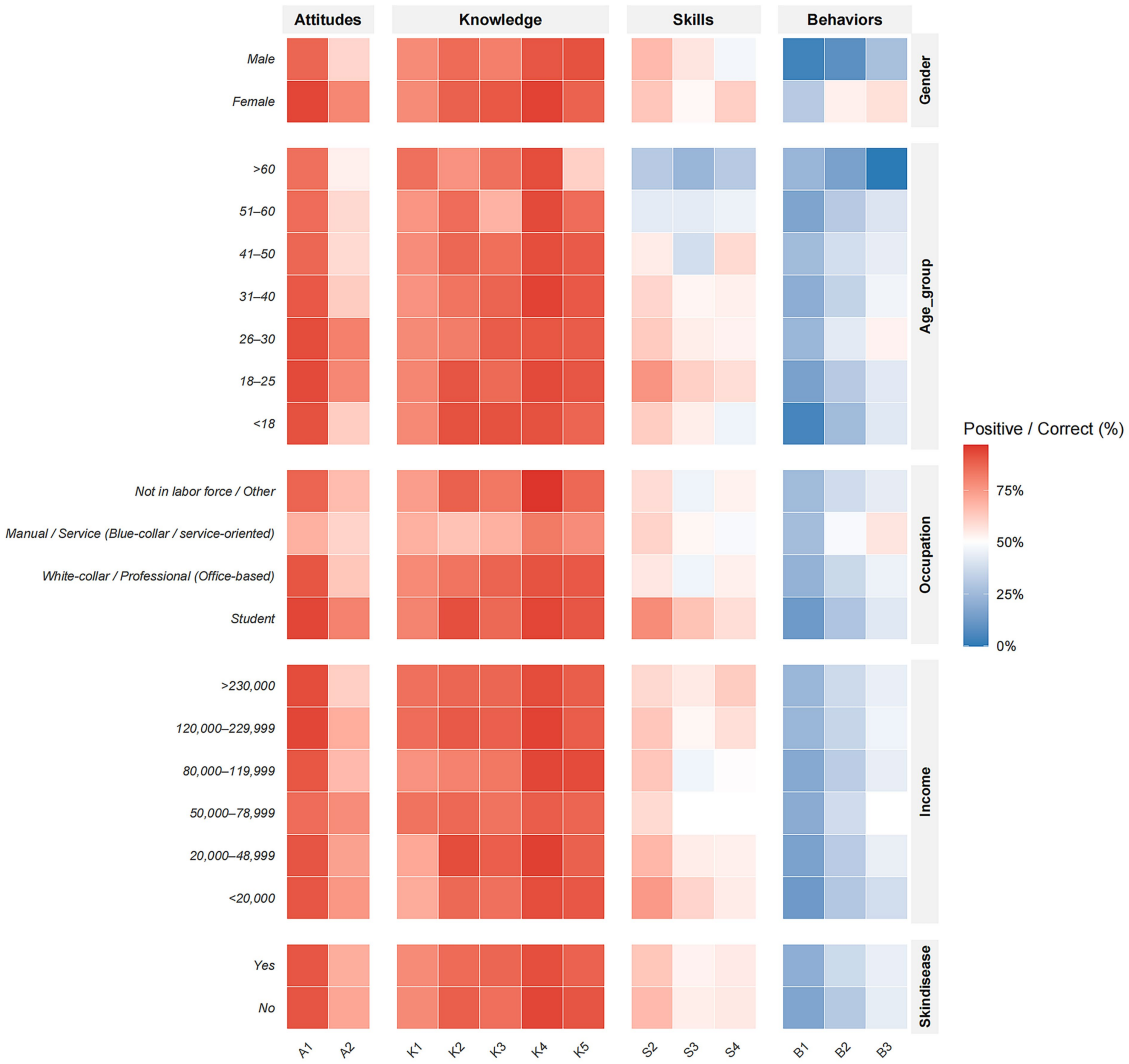
Heatmap of positive (or correct) response proportions across baseline subgroups for knowledge, attitude, self-efficacy and behavior items.

Furthermore, consistent patterns of differences across multiple items were observed among different baseline subgroups (Figure 2 and Supplementary Table S5). First, gender differences were most prominent in the behavioral items. When using “≥3” as the threshold for positive behavior, the proportion of women reporting frequent sunscreen use (B1) was 31.0% (148/478), significantly higher than that of men at 3.1% (12/387). For frequent moisturizer use (B2), the proportion was 53.8% (257/478) in women compared to 8.5% (33/387) in men. For frequent facial cleansing (B3), it was 57.5% (275/478) in women versus 26.9% (104/387) in men. Second, the self-efficacy items displayed a clear age gradient. The proportion for S2 was 76.4% (279/365) in the 18–25 age group, decreasing to 30.8% (4/13) in the >60 age group. For S3, it was 61.6% (225/365) in the 18–25 group and 23.1% (3/13) in the >60 group. Additionally, comprehension of sunscreen labels (S4) also declined with age and was higher in women than in men (62.1% vs. 46.8%). The overall accuracy rate for knowledge judgment questions was high but decreased in specific populations. The accuracy rate for K5 was only 61.5% (8/13) in the >60 group, compared to 90.7% (331/365) in the 18–25 group. Among manual/service-oriented workers, the accuracy rates for some knowledge items were relatively low. In contrast, the attitude items generally remained at a high level. However, a significant difference was observed for A2 (actively seeking information), with 79.5% (380/478) of women reporting this attitude compared to 60.5% (234/387) of men. This proportion also showed a declining trend with increasing age (79.5% in the 18–25 group; 53.9% in the >60 group).

## Discussion

Health promotion targeting community populations typically requires “measurable indicators” to identify weaknesses and evaluate intervention effectiveness. This study developed and validated a skin health knowledge, attitudes, and behaviors questionnaire covering knowledge, self-efficacy, attitudes, and behaviors. Psychometric evidence was provided from a sample of 865 individuals from the general population. Expert consultation results indicated high consistency in item relevance evaluation, with overall good content validity, suggesting that the items adequately represent the target constructs and possess a foundation for further application and dissemination.

Previous studies on skin health-related KAP have predominantly focused on specific topics [e.g., sun protection/sunscreen use or specific populations (e.g., healthcare professionals or student groups)] (18–21). The questions in these studies often emphasized general knowledge and attitudes, with insufficient coverage of competency dimensions such as “information discrimination, judgment of media/advertising credibility, and label comprehension and application.” Consequently, they were unable to systematically reflect differences in the public’s ability to process skin health information and make decisions. In contrast, while retaining key care behavior items, this study incorporated items related to misconception recognition and competencies in information acquisition, comprehension, evaluation, and application. Furthermore, by employing an IRT model, it provides item-level discrimination and threshold information, and offers more refined evidence to support scale optimization and application scenarios.

Regarding the psychometric evidence of the scale, validation was conducted at three levels. First, content validity was assessed using expert relevance ratings to calculate the CVI. This metric is commonly employed and widely recommended in scale development to evaluate whether the content adequately and appropriately represents the intended construct (22). The results based on expert review indicated that most items achieved high I_CVI_ and maintained strong agreement after correction, suggesting a solid content foundation at the level of “relevance/representativeness.” Second, for structural validity, a one-factor model was fitted to the items of the self-efficacy dimension, and indices such as CFI/TLI, RMSEA, and SRMR were reported to verify the appropriateness of its unidimensional structure (23). Finally, within the framework of IRT, a 2PL model was applied to dichotomous judgment items, and GRM was used for Likert-type ordered polytomous items (24–26). Discrimination and difficulty/threshold parameters were obtained, with the results demonstrating that these items possessed good discriminative ability and an ideal range of difficulty. Overall, the combined evidence supports that the questionnaire exhibits satisfactory content rationality and structural interpretability within the general population sample.

Analysis of item response distributions revealed that the most prominent misconception among the true/false questions was concentrated on the statement, “Human facial aging is mainly determined by genetic factors and is unrelated to lifestyle” (K1). This “genetic determinism” type of understanding may stem from the public’s intuitive inference or media narratives regarding “genes determining appearance/aging.” Previous health communication research suggests that stronger beliefs in genetic determinism are often associated with lower perceived controllability, which may consequently affect the adoption and maintenance of preventive behaviors (27). However, skin health is not solely influenced by the genetic component; it is also substantially related to environmental factors (28, 29). Therefore, this finding indicates that subsequent health education should prioritize targeted correction of the misconception that “lifestyle is not important.”

Regarding the Likert items, it was observed that the response distributions for the attitude items (A1/A2) and some basic self-efficacy items (S2) were generally skewed toward the higher score end. This suggests that respondents widely recognize the importance of skin care and possess a certain level of competence in “accessing/understanding” related information. In contrast, items involving information discrimination and application [e.g., S3: I can judge whether claims about skincare in the media or advertisements are scientifically credible; S4: I can understand the labels and instructions on sunscreen product packaging (e.g., SPF value)] exhibited a higher proportion of “uncertain” responses. This indicates that respondents have more pronounced difficulties in further evaluating, verifying, and translating information into actionable judgments. Previous research has emphasized that populations often find it easier to access information, while assessing information quality and practical application poses greater challenges (30). Concurrently, prior studies on the comprehension of sunscreen labeling have also shown that even among individuals with usage experience, significant gaps may exist in understanding label terminology and meanings (31). These findings suggest that subsequent health education and interventions should shift their focus from “enhancing awareness of importance” to “strengthening self-efficacy in evaluating health information and interpreting product labels,” aiming to reduce uncertainty and enhance decision-making quality.

For multiple-choice questions regarding ultraviolet radiation hazards, a higher proportion of respondents selected “tanning/darkening” and “photoaging,” while the selection rates for “skin cancer” and “eye injury” were relatively lower. Previous research indicates that the public is often more readily motivated by visible changes (such as photoaging) and has relatively insufficient perception and concern regarding the risks of long-term health outcomes (such as skin cancer) (32). Meanwhile, regarding factors influencing facial aging, lifestyle factors such as sleep and stress were frequently selected, suggesting that the public already possesses some intuitive understanding of the connection between “lifestyle and skin condition.” Furthermore, in terms of information acquisition channels, both doctors/pharmacists and video platforms accounted for high proportions, indicating that professional sources still hold considerable influence. Previous research has pointed out that while social media increases accessibility in skin health communication, it may also introduce unreliable information sources (33). In summary, regarding the factors related to skin damage, awareness of their long-term hazards should be enhanced, and the reliability of related information sources should also be critically evaluated.

Differences in item responses were observed across baseline subgroups. Overall, the accuracy rate for basic knowledge judgment items (K1–K5) was relatively high in most subgroups. However, significant gender differences were noted, with variations observed in some items across K-, A-, S- and B-type questions. The overall proportion of correct answers was consistently lower in men than in women. This discrepancy may be related to gender differences in proactive health information acquisition and skin protection behaviors. Previous studies have indicated that men generally exhibit lower engagement in daily attention to skin health and preventive behaviors (e.g., sun protection/skin care) and may have less exposure to and accumulation of related knowledge, leading to a higher likelihood of misjudgment or lower accuracy on questions involving lifestyle and skin care (18, 34). Additionally, differences in accuracy rates were observed across occupations, with Manual/Service (Blue-collar/service-oriented) workers showing relatively lower accuracy compared to other occupational groups, which may be associated with insufficient health literacy (35). Notably, in terms of the overall age distribution, younger age groups demonstrated higher accuracy rates, which gradually declined with increasing age. This trend may be attributed to age-related barriers in technology use and information processing, making it more difficult for older individuals to actively acquire and evaluate online health information (36).

The strength of this study lies in the systematic development and evaluation of a multidimensional skin health knowledge, attitudes, and behaviors questionnaire, facilitating structured interpretation and potential intervention planning. The study also provides a relatively comprehensive chain of psychometric evidence (expert content validity, CFA structural validity for the Self-efficacy dimension, and IRT item parameters), thereby increasing the rigor of scale development and validation. Furthermore, by unifying the scoring direction and presenting differences across various baseline subgroups using stratified proportions/heatmaps, vulnerable items and key target populations can be visually identified, providing a basis for subsequent precise health education and intervention design.

However, the study has certain limitations. First, as an online cross-sectional survey, the sample may be subject to selection bias, and the small sample size in some subgroups may affect the stability of stratified comparisons and estimates. Second, the questionnaire primarily relies on self-reported data, where behaviors and past skin disease history may be influenced by recall bias and social desirability bias. Moreover, only a single measurement was conducted, and longitudinal evidence such as test–retest reliability and predictive validity is currently lacking. Finally, structural validity was primarily verified via CFA for the Self-efficacy dimension. Due to constraints imposed by item types, conventional structural validation was not performed for the knowledge and attitude dimensions. Further validation of the scale’s structure and measurement invariance across populations in larger and more diverse samples is still required. In addition, several dimensions included a limited number of indicators, particularly the attitude dimension with only two items, which may limit the stability and interpretability of the CFA model despite the acceptable model fit.

## Conclusion

A multidimensional skin health knowledge, attitudes, and behaviors questionnaire was developed and validated in this study. Good content validity and item-level psychometric support were obtained in a community-based sample. The results indicated that population differences were primarily concentrated in self-efficacy, information acquisition and evaluation, and key skin-care behaviors such as sun protection and moisturization. Vulnerable groups included older adults, men, and individuals in manual or service-oriented occupations. This instrument can be utilized to identify key targets for intervention and to evaluate the effectiveness of health education programs. Further validation in larger and more diverse samples is warranted in the future.

## Data Availability

The original contributions presented in the study are included in the article/Supplementary material, further inquiries can be directed to the corresponding authors.
