# Supplementary material for: Development and validation of a skin health knowledge, attitudes, and behaviors questionnaire for the general population: a cross-sectional study
Source: Front Public Health. 2026 Jun 8;14:1856843. doi: 10.3389/fpubh.2026.1856843 (PMC13284075; doi:10.3389/fpubh.2026.1856843)
Supplement: Supplementary file 1 [file Data_Sheet_1.PDF]

Supplementary Appendix S1. Informed Consent Form for the Skin Health Knowledge, Attitudes, and Behaviours Questionnaire

Dear Participant,

Hello!

We are conducting a survey on public skin health knowledge, attitudes, and behaviors. The purpose of this study is to better understand public awareness of skin health–related information and daily skin-care practices. The results of this study will be used for academic research and may help improve future skin health education strategies.

This study is conducted through an anonymous online questionnaire. No directly identifiable personal information, such as names, identification numbers, or contact details, will be collected. The questionnaire contains approximately 26 items and will take about 5 minutes to complete.

Your participation is entirely voluntary. You may choose whether to participate in this study and may withdraw at any time during the survey without any negative consequences.

This study involves no foreseeable significant risks. All collected data will be used solely for research purposes and will be kept strictly confidential. The results will be reported only in aggregated form and will not disclose any personal information.

By clicking “Continue” or submitting the questionnaire, you confirm that you have read and understood the information above and voluntarily agree to participate in this study.

Thank you for your participation and support!

Supplementary Table S1. Skin Health Knowledge, Attitudes, and Behaviours Questionnaire

This questionnaire includes baseline characteristics, history of skin diseases, and items categorized as Knowledge (K), Self-efficacy (S), Attitudes (A), and Behaviors (B).

Content	Item	Question / Statement	Response options	Intended sub-dimension	Coding / notes
**Baseline information**	Baseline-1	Sex (single choice)	A. Man [ ] B. Woman [ ]	-	Nominal
	Baseline-2	Age group (single choice)	A. <18 [ ] B. 18-25 [ ] C. 26-30 [ ] D. 31-40 [ ] E. 41-50 [ ] F. 51-60 [ ] G. >60 [ ]	-	Ordinal
	Baseline-3	Occupation (single choice)	A. Student [ ] B. Government/public institution employee [ ] C. Professional (e.g., teacher/physician/lawyer) [ ] D. Service worker (e.g., tourism/catering) [ ] E. Production worker (e.g., agriculture/forestry/animal husbandry/fishery/water conservancy) [ ] F. Technical worker (e.g., manufacturing/transportation) [ ] G. Company employee [ ] H. Retired [ ] I. Self-employed/freelancer [ ]	-	Nominal
	Baseline-4	Annual household income (RMB) (single choice)	A. <20,000 [ ] B. 20,000-49,999 [ ] C. 50,000-79,999 [ ] D. 80,000-119,999 [ ] E. 120,000-230,000 [ ] F. >230,000 [ ]	-	Ordinal
**History of skin diseases (Baseline)**	History-1	Have you ever been diagnosed by a doctor with any of the following skin	A. Eczema [ ] B. Urticaria [ ] C. Acne [ ] D. Hair loss (cause unspecified) [ ] E.Hair loss (Androgenetic) F. Alopecia areata [ ] G. Vitiligo [ ] H. Psoriasis [ ]	-	Multiple responses allowed; each option can be coded 0/1

		diseases? (multiple choice)	I. Contact dermatitis [ ] J. Rosacea [ ] K. Herpes zoster [ ] L. Scabies [ ] M. Viral warts (e.g., common/plantar/flat warts) [ ] N. Tinea pedis or tinea corporis (fungal infection) [ ] O. Other (specify: ____) [ ]		
**Knowledge (K1-K5)(True/False)**	K1	Human facial aging is mainly determined by genetic factors and is unrelated to lifestyle.	A. Yes [ ] B. No [ ]	Lifestyle-related skin aging knowledge	True/False item
	K2	There is a saying that "drinking alcohol can improve beauty/complexion," so people who drink alcohol have better skin.	A. Yes [ ] B. No [ ]	Misconception awareness regarding alcohol and skin health	True/False item
	K3	Smokers' facial skin ages faster.	A. Yes [ ] B. No [ ]	Smoking-related skin aging knowledge	True/False item
	K4	Regular exercise makes people look younger.	A. Yes [ ] B. No [ ]	Lifestyle and exercise-related skin health knowledge	True/False item
	K5	The more expensive the skincare products, the better the results.	A. Yes [ ] B. No [ ]	Skincare misconception awareness	True/False item

	QC-K	Quality control item (for response quality assessment): Please select “No”.	A. Yes [ ] B. No [ ]	-	Data quality check; responses not following instruction can be flagged
**Knowledge(K6-K7 (Likert; misconception awareness; reverse-coded knowledge belief))**	K6	When I have acne (pimples) on my face, skincare products can cure it.	A. Strongly agree [ ] B. Agree [ ] C. Not sure [ ] D. Disagree [ ] E. Strongly disagree [ ]	Skin care misconception awarenes	Likert; reverse-coded (misconception item)
	K7	When my skin is sunburned or becomes red from sun exposure, skincare products can cure it.	A. Strongly agree [ ] B. Agree [ ] C. Not sure [ ] D. Disagree [ ] E. Strongly disagree [ ]	Sun exposure and skin care misconception awareness	Likert; reverse-coded (misconception item)
**Knowledge(K8-K10 (Multiple choice; application of knowledge))**	K8	Which of the following do you think can influence facial aging? (Select all that apply.)	A. Stress [ ] B. Hormonal changes [ ] C. Sleep [ ] D. Exercise [ ] E. Smoking [ ] F. Alcohol drinking [ ] G. Diet [ ] H. Sun protection [ ] I. Skincare [ ] J. Skin type [ ] K. Other (specify: ____) [ ]	Knowledge of factors influencing facial aging	Multiple responses allowed; each option can be coded 0/1
	K9	What do you think is the main harm of ultraviolet (UV) radiation to the human body?	A. Skin darkening/tanning [ ] B. Skin aging [ ] C. Skin cancer [ ] D. Eye damage [ ]	UV-related skin health knowledge	Multiple responses allowed; each option can be coded 0/1
	K10	Which workplace environmental exposures do you think may affect skin health? (Select all that apply.)	A. Chemicals [ ] B. Dust [ ] C. High temperature [ ] D. Sun exposure [ ] E. Microorganisms [ ] F. Friction and pressure [ ]	Occupational skin health knowledge	Multiple responses allowed; each option can be coded 0/1

**Self-efficacy (S)**	S1	I can understand doctors' advice for daily life, such as healthy eating and going to bed on time.	A. Strongly agree (4) [ ] B. Agree (3) [ ] C. Not sure (2) [ ] D. Disagree (1) [ ] E. Strongly disagree (0) [ ]	Understanding health information	4-0 points
	S2	I can find reliable skin health information on the internet.	A. Strongly agree (4) [ ] B. Agree (3) [ ] C. Not sure (2) [ ] D. Disagree (1) [ ] E. Strongly disagree (0) [ ]	Information acquisition	4-0 points
	S3	I can judge whether claims about skincare in the media or advertisements are scientifically credible.	A. Strongly agree (4) [ ] B. Agree (3) [ ] C. Not sure (2) [ ] D. Disagree (1) [ ] E. Strongly disagree (0) [ ]	Information evaluation	4-0 points
	S4	I can understand the labels and instructions on sunscreen product packaging (e.g., SPF value).	A. Strongly agree (4) [ ] B. Agree (3) [ ] C. Not sure (2) [ ] D. Disagree (1) [ ] E. Strongly disagree (0) [ ]	Information interpretation	4-0 points
**Attitudes (A)**	A1	I know the importance of skincare.	A. Strongly agree (4) [ ] B. Agree (3) [ ] C. Not sure (2) [ ] D. Disagree (1) [ ] E. Strongly disagree (0) [ ]	Perceived importance of skin care	4-0 points
	A2	I actively look for information about skin health and skincare.	A. Strongly agree (4) [ ] B. Agree (3) [ ] C. Not sure (2) [ ] D. Disagree (1) [ ] E. Strongly disagree (0) [ ]	Proactive attitude toward skin health information	4-0 points

**Behaviors (B)(B1-B4 (Skin health-related behaviors in the past year))**	B1	Frequency of UV protection when going out on sunny summer days (single choice)	A. Never [ ] B. 1-2 times/week [ ] C. 3-5 times/week [ ] D. Almost every day [ ]	Sun protection behaviour	Ordinal; can code 1-4
	B2	Frequency of using daily skincare products with moisturizing/hydrating functions (single choice)	A. Never [ ] B. 1-2 times/week [ ] C. 3-5 times/week [ ] D. Almost every day [ ] E. >=2 times/day [ ]	Moisturization behaviour	Ordinal; can code 1-5
	B3	Frequency of using facial cleansing products (e.g., facial cleanser/cleansing soap) (single choice)	A. Never [ ] B. 1-2 times/week [ ] C. 3-5 times/week [ ] D. Almost every day [ ] E. >=2 times/day [ ]	Cleansing behaviour	Ordinal; can code 1-5
	B4	Frequency of using moisturizing/hydrating face masks (single choice)	A. Almost never [ ] B. <1 time/month [ ] C. 1-2 times/month [ ] D. >2 times/month [ ] E. Random/irregular [ ]	Skin care maintenance behaviour	Ordinal/other; "Random" may be treated as separate category
**Behaviors (B)(B5 (Information sources; classified under Behaviors))**	B5	Through which channels do you usually obtain skin health-related information? (multiple choice)	A. Doctors/pharmacists [ ] B. Friends/family [ ] C. Official accounts (e.g., WeChat public accounts) [ ] D. Videos [ ] E. Apps [ ] F. Magazines [ ] G. Other (specify: ____) [ ]	Information-seeking behaviour	Multiple responses allowed; each option can be coded 0/1

Note: Items K6-K7 are misconception awareness statements and are typically reverse-coded in scoring, depending on the study design. QC-K is a quality control item.

Table S2 . Content validity indices (CVI) for questionnaire items (n = 13 experts).

Item code	I_{CVI}	Pc	K*
In the past year, how often have you used facial cleansing products (e.g., facial cleanser, cleansing soap)?	1.000	0.00012	1.000
In the past year, how often have you used moisturizing/hydrating sheet masks (or masks with moisturizing effects)?	1.000	0.00012	1.000
Human facial aging is mainly determined by genetic factors and is unrelated to lifestyle.	1.000	0.00012	1.000
There is a saying that “drinking alcohol can improve beauty/complexion,” so people who drink alcohol have better skin.	1.000	0.00012	1.000
Smokers’ facial skin ages faster.	1.000	0.00012	1.000
Regular exercise makes people look younger	1.000	0.00012	1.000
I actively look for information about skin health and skincare.	1.000	0.00012	1.000
When my skin is sunburned or becomes red from sun exposure, skincare products can cure it.	1.000	0.00012	1.000
I can understand the labels and instructions on sunscreen product packaging (e.g., SPF value).	1.000	0.00012	1.000
What do you think is the main harm of ultraviolet (UV) radiation to the human body?	1.000	0.00012	1.000
In the past year, how often have you used daily skincare products with moisturizing/hydrating effects?	0.923	0.00159	0.923

Which of the following do you think can influence facial aging? (Select all that apply.)	0.923	0.00159	0.923
In the past year, how often did you use UV protection (e.g., sunscreen, umbrella, hat, sun-protective clothing) when going outdoors on sunny summer days?	0.846	0.00952	0.845

Notes. I_{CVI} = item-level content validity index. Pc = probability of chance agreement. K* = modified kappa correcting for chance agreement. Ratings were dichotomized as relevant (3–4) vs not relevant (1–2). n = 13 experts.

Table S3. Standardized factor loadings from CFA

Factor	Item	SE	z	p	λ
Attitudes	A1	0.022	34.031	<0.001	0.747
Attitudes	A2	0.016	54.337	<0.001	0.886
Behaviors	B1	0.023	31.668	<0.001	0.736
Behaviors	B2	0.023	42.211	<0.001	0.961
Behaviors	B3	0.023	30.979	<0.001	0.721
Knowledge_Factual	K1	0.071	5.312	<0.001	0.378
Knowledge_Factual	K2	0.074	5.527	<0.001	0.411
Knowledge_Factual	K3	0.078	9.403	<0.001	0.730
Knowledge_Factual	K4	0.081	8.270	<0.001	0.671
Knowledge_Factual	K5	0.089	1.819	0.069	0.162
Knowledge_Misconception	K6	0.035	24.402	<0.001	0.847
Knowledge_Misconception	K7	0.033	24.164	<0.001	0.789
Self-efficacy	S1	0.031	20.429	<0.001	0.637
Self-efficacy	S2	0.015	54.681	<0.001	0.816
Self-efficacy	S3	0.020	37.311	<0.001	0.737
Self-efficacy	S4	0.025	23.699	<0.001	0.598

Note. CFA was estimated in lavaan using WLSMV with ordered categorical indicators (N=865). A five-factor model was specified, splitting Knowledge into two correlated factors (Knowledge_Factual: K1–K5; Knowledge_Misconception: K6–K7) alongside Self-efficacy (S1–S4), Attitudes (A1–A2), and Behaviors (B1–B3). Model fit: $\chi^2(94)=494.34$, $p<0.001$; CFI=0.969; TLI=0.960; RMSEA=0.070; SRMR=0.095. λ denotes standardized factor loadings.

Table S4. Responses to skin health literacy statements (Yes/No), N=865

Statement	Correct answer	Correct, n (%)	Incorrect (misconception), n (%)
**Facial aging is mainly determined by genetics and unrelated to lifestyle.**	No	680 (78.6)	185 (21.4)
**"Alcohol improves skin," so drinkers have better skin.**	No	755 (87.3)	110 (12.7)
**Smokers' facial skin ages faster.**	Yes	745 (86.1)	120 (13.9)
**Regular exercise makes people look younger.**	Yes	803 (92.8)	62 (7.2)
**The more expensive the skincare products, the better the effect.**	No	774 (89.5)	91 (10.5)

Table S5. Positive/correct response proportions by baseline subgroups

Level	BaselineVar	Item	Positive (≥3) / Correct, n/N (%)
Female	Gender	A1	449/478(93.9)
Male	Gender	A1	337/387(87.1)
<18	Age_group	A1	22/24(91.7)
18–25	Age_group	A1	339/365(92.9)
26–30	Age_group	A1	87/94(92.6)
31–40	Age_group	A1	191/212(90.1)
41–50	Age_group	A1	100/115(87.0)
51–60	Age_group	A1	36/42(85.7)
>60	Age_group	A1	11/13(84.6)
Student	Occupation	A1	326/348(93.7)
White-collar / Professional (Office-based)	Occupation	A1	339/374(90.6)
Manual / Service (Blue-collar / service-oriented)	Occupation	A1	16/23(69.6)
Not in labor force / Other	Occupation	A1	105/120(87.5)
<20,000	Income	A1	168/185(90.8)
20,000–48,999	Income	A1	122/134(91.0)
50,000–78,999	Income	A1	103/120(85.8)
80,000–119,999	Income	A1	113/125(90.4)
120,000–229,999	Income	A1	129/138(93.5)
>230,000	Income	A1	151/163(92.6)
No	Skindisease	A1	386/424(91.0)
Yes	Skindisease	A1	400/441(90.7)
Female	Gender	A2	380/478(79.5)
Male	Gender	A2	234/387(60.5)
<18	Age_group	A2	15/24(62.5)

18–25	Age_group	A2	290/365(79.5)
26–30	Age_group	A2	76/94(80.9)
31–40	Age_group	A2	133/212(62.7)
41–50	Age_group	A2	68/115(59.1)
51–60	Age_group	A2	25/42(59.5)
>60	Age_group	A2	7/13(53.9)
Student	Occupation	A2	281/348(80.8)
White-collar / Professional (Office-based)	Occupation	A2	239/374(63.9)
Manual / Service (Blue-collar / service-oriented)	Occupation	A2	14/23(60.9)
Not in labor force / Other	Occupation	A2	80/120(66.7)
<20,000	Income	A2	140/185(75.7)
20,000–48,999	Income	A2	98/134(73.1)
50,000–78,999	Income	A2	94/120(78.3)
80,000–119,999	Income	A2	84/125(67.2)
120,000–229,999	Income	A2	97/138(70.3)
>230,000	Income	A2	101/163(62.0)
No	Skindisease	A2	305/424(71.9)
Yes	Skindisease	A2	309/441(70.1)
Female	Gender	B1	148/478(31.0)
Male	Gender	B1	12/387(3.1)
<18	Age_group	B1	1/24(4.2)
18–25	Age_group	B1	56/365(15.3)
26–30	Age_group	B1	22/94(23.4)
31–40	Age_group	B1	42/212(19.8)
41–50	Age_group	B1	29/115(25.2)
51–60	Age_group	B1	7/42(16.7)

>60	Age_group	B1	3/13(23.1)
Student	Occupation	B1	43/348(12.4)
White-collar / Professional (Office-based)	Occupation	B1	81/374(21.7)
Manual / Service (Blue-collar / service-oriented)	Occupation	B1	6/23(26.1)
Not in labor force / Other	Occupation	B1	30/120(25.0)
<20,000	Income	B1	23/185(12.4)
20,000–48,999	Income	B1	21/134(15.7)
50,000–78,999	Income	B1	23/120(19.2)
80,000–119,999	Income	B1	23/125(18.4)
120,000–229,999	Income	B1	32/138(23.2)
>230,000	Income	B1	38/163(23.3)
No	Skindisease	B1	71/424(16.8)
Yes	Skindisease	B1	89/441(20.2)
Female	Gender	B2	257/478(53.8)
Male	Gender	B2	33/387(8.5)
<18	Age_group	B2	6/24(25.0)
18–25	Age_group	B2	113/365(31.0)
26–30	Age_group	B2	40/94(42.6)
31–40	Age_group	B2	72/212(34.0)
41–50	Age_group	B2	44/115(38.3)
51–60	Age_group	B2	13/42(31.0)
>60	Age_group	B2	2/13(15.4)
Student	Occupation	B2	100/348(28.8)
White-collar / Professional (Office-based)	Occupation	B2	134/374(35.8)
Manual / Service (Blue-collar / service-oriented)	Occupation	B2	11/23(47.8)
Not in labor force / Other	Occupation	B2	45/120(37.5)

<20,000	Income	B2	55/185(29.7)
20,000–48,999	Income	B2	42/134(31.3)
50,000–78,999	Income	B2	45/120(37.5)
80,000–119,999	Income	B2	40/125(32.0)
120,000–229,999	Income	B2	48/138(34.8)
>230,000	Income	B2	60/163(36.8)
No	Skindisease	B2	129/424(30.4)
Yes	Skindisease	B2	161/441(36.5)
Female	Gender	B3	275/478(57.5)
Male	Gender	B3	104/387(26.9)
<18	Age_group	B3	10/24(41.7)
18–25	Age_group	B3	154/365(42.2)
26–30	Age_group	B3	50/94(53.2)
31–40	Age_group	B3	98/212(46.2)
41–50	Age_group	B3	50/115(43.5)
51–60	Age_group	B3	17/42(40.5)
>60	Age_group	B3	0/13(0)
Student	Occupation	B3	145/348(41.7)
White-collar / Professional (Office-based)	Occupation	B3	169/374(45.2)
Manual / Service (Blue-collar / service-oriented)	Occupation	B3	13/23(56.5)
Not in labor force / Other	Occupation	B3	52/120(43.3)
<20,000	Income	B3	70/185(37.8)
20,000–48,999	Income	B3	59/134(44.0)
50,000–78,999	Income	B3	60/120(50.0)
80,000–119,999	Income	B3	55/125(44.0)
120,000–229,999	Income	B3	63/138(45.7)

>230,000	Income	B3	72/163(44.2)
No	Skindisease	B3	184/424(43.4.)
Yes	Skindisease	B3	195/441(44.2)
Female	Gender	K1	376/478(78.7)
Male	Gender	K1	304/387(78.6)
<18	Age_group	K1	19/24(79.2)
18–25	Age_group	K1	291/365(79.7)
26–30	Age_group	K1	74/94(78.7)
31–40	Age_group	K1	163/212(76.9)
41–50	Age_group	K1	90/115(78.3)
51–60	Age_group	K1	32/42(76.2)
>60	Age_group	K1	11/13(84.6)
Student	Occupation	K1	280/348(80.5)
White-collar / Professional (Office-based)	Occupation	K1	295/374(78.9)
Manual / Service (Blue-collar / service-oriented)	Occupation	K1	16/23(69.6)
Not in labor force / Other	Occupation	K1	89/120(74.2)
<20,000	Income	K1	131/185(70.8)
20,000–48,999	Income	K1	96/134(71.6)
50,000–78,999	Income	K1	101/120(84.2)
80,000–119,999	Income	K1	96/125(76.8)
120,000–229,999	Income	K1	118/138(85.5)
>230,000	Income	K1	138/163(84.7)
No	Skindisease	K1	334/424(78.8)
Yes	Skindisease	K1	346/441(78.5)
Female	Gender	K2	422/478(88.3)
Male	Gender	K2	333/387(86.1)

<18	Age_group	K2	22/24(91.7)
18–25	Age_group	K2	332/365(91.0)
26–30	Age_group	K2	77/94(81.9)
31–40	Age_group	K2	178/212(84.0)
41–50	Age_group	K2	100/115(87.0)
51–60	Age_group	K2	36/42(85.7)
>60	Age_group	K2	10/13(76.9)
Student	Occupation	K2	320/348(92.0)
White-collar / Professional (Office-based)	Occupation	K2	314/374(84.0)
Manual / Service (Blue-collar / service-oriented)	Occupation	K2	15/23(65.2)
Not in labor force / Other	Occupation	K2	106/120(88.3)
<20,000	Income	K2	160/185(86.5)
20,000–48,999	Income	K2	124/134(92.5)
50,000–78,999	Income	K2	104/120(86.7)
80,000–119,999	Income	K2	101/125(80.8)
120,000–229,999	Income	K2	124/138(89.9)
>230,000	Income	K2	142/163(87.1)
No	Skindisease	K2	376/424(88.7)
Yes	Skindisease	K2	379/441(85.9)
Female	Gender	K3	431/478(90.2)
Male	Gender	K3	314/387(81.1)
<18	Age_group	K3	22/24(91.7)
18–25	Age_group	K3	315/365(86.3)
26–30	Age_group	K3	84/94(89.4)
31–40	Age_group	K3	186/212(87.7)
41–50	Age_group	K3	98/115(85.2)

51–60	Age_group	K3	29/42(69.1)
>60	Age_group	K3	11/13(84.6)
Student	Occupation	K3	301/348(86.5)
White-collar / Professional (Office-based)	Occupation	K3	328/374(87.7)
Manual / Service (Blue-collar / service-oriented)	Occupation	K3	16/23(69.6)
Not in labor force / Other	Occupation	K3	100/120(83.3)
<20,000	Income	K3	157/185(84.9)
20,000–48,999	Income	K3	119/134(88.8)
50,000–78,999	Income	K3	101/120(84.2)
80,000–119,999	Income	K3	104/125(83.2)
120,000–229,999	Income	K3	122/138(88.4)
>230,000	Income	K3	142/163(87.1)
No	Skindisease	K3	361/424(85.1)
Yes	Skindisease	K3	384/441(87.1)
Female	Gender	K4	452/478(94.6)
Male	Gender	K4	351/387(90.7)
<18	Age_group	K4	22/24(91.7)
18–25	Age_group	K4	339/365(92.9)
26–30	Age_group	K4	85/94(90.4)
31–40	Age_group	K4	200/212(94.3)
41–50	Age_group	K4	106/115(92.2)
51–60	Age_group	K4	39/42(92.9)
>60	Age_group	K4	12/13(92.3)
Student	Occupation	K4	327/348(94.0)
White-collar / Professional (Office-based)	Occupation	K4	341/374(91.2)
Manual / Service (Blue-collar / service-oriented)	Occupation	K4	19/23(82.6)

Not in labor force / Other	Occupation	K4	116/120(96.7)
<20,000	Income	K4	171/185(92.4)
20,000–48,999	Income	K4	127/134(94.8)
50,000–78,999	Income	K4	107/120(89.2)
80,000–119,999	Income	K4	117/125(93.6)
120,000–229,999	Income	K4	130/138(94.2)
>230,000	Income	K4	151/163(92.6)
No	Skindisease	K4	397/424(93.6)
Yes	Skindisease	K4	406/441(92.1)
Female	Gender	K5	420/478(87.9)
Male	Gender	K5	354/387(91.5)
<18	Age_group	K5	21/24(87.5)
18–25	Age_group	K5	331/365(90.7)
26–30	Age_group	K5	84/94(89.4)
31–40	Age_group	K5	191/212(90.1)
41–50	Age_group	K5	103/115(89.6)
51–60	Age_group	K5	36/42(85.7)
>60	Age_group	K5	8/13(61.5)
Student	Occupation	K5	315/348(90.5)
White-collar / Professional (Office-based)	Occupation	K5	337/374(90.1)
Manual / Service (Blue-collar / service-oriented)	Occupation	K5	18/23(78.3)
Not in labor force / Other	Occupation	K5	104/120(86.7)
<20,000	Income	K5	167/185(90.3)
20,000–48,999	Income	K5	118/134(88.1)
50,000–78,999	Income	K5	105/120(87.5)
80,000–119,999	Income	K5	116/125(92.8)

120,000–229,999	Income	K5	123/138(89.1)
>230,000	Income	K5	145/163(89.0)
No	Skindisease	K5	386/424(91.0)
Yes	Skindisease	K5	388/441(88.0)
Female	Gender	S2	306/478(64.0)
Male	Gender	S2	260/387(67.2)
<18	Age_group	S2	15/24(62.5)
18–25	Age_group	S2	279/365(76.4)
26–30	Age_group	S2	59/94(62.8)
31–40	Age_group	S2	128/212(60.4)
41–50	Age_group	S2	63/115(54.8)
51–60	Age_group	S2	18/42(42.9)
>60	Age_group	S2	4/13(30.8)
Student	Occupation	S2	273/348(78.5)
White-collar / Professional (Office-based)	Occupation	S2	209/374(55.9)
Manual / Service (Blue-collar / service-oriented)	Occupation	S2	14/23(60.9)
Not in labor force / Other	Occupation	S2	70/120(58.3)
<20,000	Income	S2	139/185(75.1)
20,000–48,999	Income	S2	91/134(67.9)
50,000–78,999	Income	S2	71/120(59.2)
80,000–119,999	Income	S2	80/125(64.0)
120,000–229,999	Income	S2	88/138(63.8)
>230,000	Income	S2	97/163(59.5)
No	Skindisease	S2	284/424(67.0)
Yes	Skindisease	S2	282/441(64.0)
Female	Gender	S3	247/478(51.7)

Male	Gender	S3	218/387(56.3)
<18	Age_group	S3	13/24(54.2)
18–25	Age_group	S3	225/365(61.6)
26–30	Age_group	S3	51/94(54.3)
31–40	Age_group	S3	111/212(52.4)
41–50	Age_group	S3	44/115(38.3)
51–60	Age_group	S3	18/42(42.9)
>60	Age_group	S3	3/13(23.1)
Student	Occupation	S3	226/348(64.9)
White-collar / Professional (Office-based)	Occupation	S3	172/374(46.0)
Manual / Service (Blue-collar / service-oriented)	Occupation	S3	12/23(52.2)
Not in labor force / Other	Occupation	S3	55/120(45.8)
<20,000	Income	S3	112/185(60.5)
20,000–48,999	Income	S3	73/134(54.5)
50,000–78,999	Income	S3	60/120(50.0)
80,000–119,999	Income	S3	58/125(46.4)
120,000–229,999	Income	S3	72/138(52.2)
>230,000	Income	S3	90/163(55.2)
No	Skindisease	S3	230/424(54.3)
Yes	Skindisease	S3	235/441(53.3)
Female	Gender	S4	297/478(62.1)
Male	Gender	S4	181/387(46.8)
<18	Age_group	S4	11/24(45.8)
18–25	Age_group	S4	212/365(58.1)
26–30	Age_group	S4	50/94(53.2)
31–40	Age_group	S4	114/212(53.8)

41–50	Age_group	S4	68/115(59.1)
51–60	Age_group	S4	19/42(45.2)
>60	Age_group	S4	4/13(30.8)
Student	Occupation	S4	202/348(58.1)
White-collar / Professional (Office-based)	Occupation	S4	201/374(53.7)
Manual / Service (Blue-collar / service-oriented)	Occupation	S4	11/23(47.8)
Not in labor force / Other	Occupation	S4	64/120(53.3)
<20,000	Income	S4	101/185(54.6)
20,000–48,999	Income	S4	72/134(53.7)
50,000–78,999	Income	S4	60/120(50.0)
80,000–119,999	Income	S4	63/125(50.4)
120,000–229,999	Income	S4	80/138(58.0)
>230,000	Income	S4	102/163(62.6)
No	Skindisease	S4	235/424(55.4)
Yes	Skindisease	S4	243/441(55.1)

Note. Values are presented as n/N (%), where n is the number of respondents with a positive/correct response and N is the number of valid responses within each subgroup. For ordinal items (Attitudes A1–A2, Self-efficacy S2–S4, Behaviors B1–B3), a positive response was defined as a score ≥3. For dichotomous knowledge items (K1–K5), a correct response was defined as 1. Items K6, K7, and S1 were excluded from the final questionnaire and are not shown.
